# Left–Right Reversal Recurrently Evolved Regardless of *Diaphanous-Related Formin* Gene Duplication or Loss in Snails

**DOI:** 10.1007/s00239-023-10130-3

**Published:** 2023-09-25

**Authors:** Takeshi Noda, Noriyuki Satoh, Edmund Gittenberger, Takahiro Asami

**Affiliations:** 1 Department of Biology, Faculty of Science, Shinshu University, Matsumoto, Japan; 2 Marine Genomics Unit, Okinawa Institute of Science and Technology Graduate University, Onna, Okinawa Japan; 3 Naturalis Biodiversity Center, Leiden, Netherlands; 4 GiMaRIS, Sassenheim, Netherlands

**Keywords:** Asymmetry, Internal selection, Spiral cleavage, Gastropoda, Spiralia, Bilateria

## Abstract

Bilateria exhibit whole-body handedness in internal structure. This left–right polarity is evolutionarily conserved with virtually no reversed extant lineage, except in molluscan Gastropoda. Phylogenetically independent snail groups contain both clockwise-coiled (dextral) and counterclockwise-coiled (sinistral) taxa that are reversed from each other in bilateral handedness as well as in coiling direction. Within freshwater Hygrophila, *Lymnaea* with derived dextrality have *diaphanous related formin* (*diaph*) gene duplicates, while basal sinistral groups possess one *diaph* gene. In terrestrial Stylommatophora, dextral *Bradybaena* also have *diaph* duplicates. Defective maternal expression of one of those duplicates gives rise to sinistral hatchlings in *Lymnaea* and handedness-mixed broods in *Bradybaena*, through polarity change in spiral cleavage of embryos. These findings led to the hypothesis that *diaph* duplication was crucial for the evolution of dextrality by reversal. The present study discovered that *diaph* duplication independently occurred four times and its duplicate became lost twice in gastropods. The dextrality of *Bradybaena* represents the ancestral handedness conserved across gastropods, unlike the derived dextrality of *Lymnaea*. Sinistral lineages recurrently evolved by reversal regardless of whether *diaph* had been duplicated. Amongst the seven *formin* gene subfamilies, *diaph* has most thoroughly been conserved across eukaryotes of the 14 metazoan phyla and choanoflagellate. Severe embryonic mortalities resulting from insufficient expression of the duplicate in both of *Bradybaena* and *Lymnaea* also support that *diaph* duplicates bare general roles for cytoskeletal dynamics other than controlling spiralian handedness. Our study rules out the possibility that *diaph* duplication or loss played a primary role for reversal evolution.

**Supplementary Information:**

The online version contains supplementary material available at 10.1007/s00239-023-10130-3.

## Introduction

Bilateria possess a characteristic body plan with the left–right axis. Their body architecture may externally be left–right symmetric by and large. On the other hand, their internal structure frequently exhibits bilateral asymmetry such as seen in the organ arrangement. In this whole-body asymmetry, virtually no left–right reversed taxa are known (Wood 2005), indicating the presence of evolutionary constraint against reversal across bilaterian groups except for one: molluscan gastropods. Diverse snail groups with internal fertilization uniquely present both clockwise-coiled (dextral) and counterclockwise-coiled (sinistral) taxa in many phylogenetically independent clades (Vermeij 1975; Okumura et al. 2008; Ponder et al. 2020a).

**Fig. 1:**
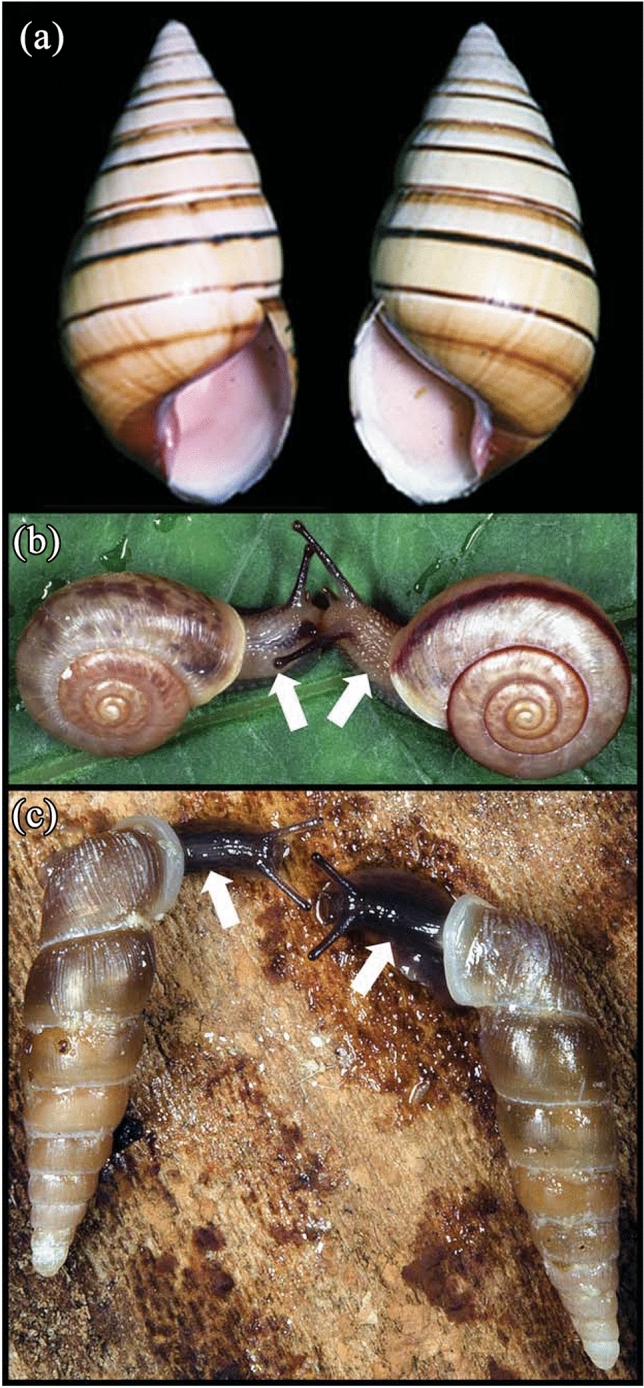
Left–right handedness of gastropod snails in coiling direction (secondary asymmetry) and bilateral (primary) asymmetry. **a** Dextral (left in figure) and sinistral shells of a chirally dimorphic Cuban tree snail *Liguus vittatus*. **b** Dextral (left) and sinistral siblings produced by a racemic mutant parent in a dextral Asian terrestrial snail *Bradybaena similaris*. **c** Dextral (left) and sinistral siblings produced by a racemic mutant parent in a sinistral Asian clausiliid terrestrial snail *Euphaedusa tau*. Arrows indicate genital orifice locations reversed between the dextral and sinistral

The aperture of the dextral shell is to the right when the apex is up and to the left in sinistral snails (Fig. 1a). In most gastropod groups, their coiling direction reflects the polarity of bilateral asymmetry in the body plan of Bilateria (Ponder et al. 2020a). The location of their genital orifice, situated at the body side instead of the midplane unlike typical bilaterians, is thus also reversed between dextral and sinistral gastropods (Fig. 1b, c). Therefore, physical genital mismatch interferes with copulation between the reversed mutant and the wild-type, resulting in positive frequency-dependent selection against reversal in gastropods with internal fertilization (Johnson 1982; Gittenberger 1988; Asami et al. 1998). Because of this mechanism, reversed individuals are rarely found in wild populations. Their lineages/groups mostly remain without population fixation for the reversal, i.e., reversal evolution. Reversed taxa are unknown in marine gastropods with external fertilization, in which stringent survival selections against reversal can be in effect under large effective population size (Vermeij 1975; Utsuno et al. 2011).

Typical difficulties of gastropod breeding in captivity with rarely available mutants and a characteristic inheritance mode, maternal inheritance (Toyama 1913; Sturtevant 1923), historically hampered studies to determine the genetic basis of gastropod handedness (Boycott and Diver 1923; Boycott et al. 1930). Most results of crossing experiment supported inheritance of whole-body handedness at one nuclear locus in four phylogenetically independent groups of hermaphroditic pulmonates (Murray and Clarke 1976; Asami et al. 2008; Utsuno and Asami 2010; Utsuno et al. 2010). It deserves attention, however, that the wild-type handedness was contrasted with the mutant phenotype in most cases. At least in freshwater pulmonate (hygrophilid) snails, early spiral cleavages proceed in largely opposite direction between dextral and sinistral taxa (Crampton 1894; Camey and Verdonk 1970; Raven 1972; Meshcheryakov and Beloussov 1975). This reversal of spiral cleavage in early development between the wild types with opposite handedness, however, remains unverified in other gastropod clades, for similar technical reasons that hinder breeding.

A racemic mutant line of terrestrial dextral *Bradybaena similaris* produces handedness-mixed broods from which both dextral and sinistral progeny consistently hatch (Fig. 1b, Utsuno and Asami 2010). Another racemic mutant strain has been established in sinistral clausiliid *Euphaedusa tau* (Fig. 1c, Utsuno et al. 2010). Similarly mixed broods were observed in crossing experiments with dextral mutants of sinistral *Alinda biplicata* (Degner 1952) and in chirally dimorphic *Partula* snails (Murray and Clarke 1976). In freshwater gastropods, handedness-mixed broods were also found to be produced by genetically fixed strains of dextral *Lymnaea peregra* (Freeman and Lundelius 1982) as well as in wild populations of an operculate dextral *Campeloma* (van Cleave 1936). These findings illuminate the persistence of an evolutionarily conserved genetic system for development of the wild-type handedness, for which gastropod populations are ordinarily fixed to be either dextral or sinistral (Hierck et al. 2005; Okumura et al. 2008; Utsuno et al. 2011).

Bilaterally reversed gastropods evolved through population fixation for the reversal in this conserved developmental system, in which early embryos undergo spiral cleavages (Lesoway and Henry 2019; Martín-Durán and Marlétaz 2020). This evolutionary process has been accelerated through the course of lineage radiation in the land pulmonate clade Stylommatophora. The gene responsible for the reversal has acted as a speciation gene that establishes sexual isolation by reversal through population fixation under given conditions of terrestrial environment (Gittenberger 1988; Orr 1991; van Batenburg and Gittenberger 1996; Ueshima and Asami 2003; Hoso et al. 2010; Richards et al. 2017). The frequency of chiral speciation was estimated as once every 4.6–3.3 MY in the door snail family Clausiliidae (Gittenberger et al. 2012).

In the case of a handedness mutant strain of *L. stagnalis*, established with reversed (sinistral) individuals found in a natural pond (Hierck et al. 2005; Asami et al. 2008), the sinistral phenotype of hatching individuals was the outcome of frameshift mutation at one of the *diaphanous-related formin* (*diaph*) gene duplicates (Davison et al. 2016; Kuroda et al. 2016). Insufficient expression of the *diaph* duplicate results in loss of maternal handedness determination in the racemic strain of terrestrial *B. similaris*, which thus produces handedness-mixed broods (Noda et al. 2019). These cases indicate that the *diaph* duplicate bares an indispensable role for development of the wild-type dextrality at least in pulmonate clades.

Within a freshwater clade, composed of Lymnaeidae and two sinistral families Physidae and Planorbidae, duplication of the *diaph* gene probably occurred in the ancestor of Lymnaeidae, because other sinistral groups have a single *diaph* gene in genome (Davison et al. 2016). Dextral *B. similaris* of the terrestrial stylommatophoran clade also possesses *diaph* duplicates (Noda et al. 2019). Whether recurrent reversal evolution occurred depending on *diaph* duplication has, therefore, arisen as a critical question to be answered. However, few studies examined how the molecular evolution of *diaph* proceeded during the course of gastropod cladogenesis. It thus remains unknown whether the wild-type sinistrality of those reversed lineages could have originated in the evolutionary change of *diaph*. Here, we show that the reversal of bilateral handedness recurrently evolved without depending on the dynamic molecular evolution of *diaph* that occurred in Gastropoda.

## Methods

### Molluscan *Diaph* Sequences

*Diaph* sequences of molluscan species were assembled using available RNA sequence reads and whole-genome sequences deposited in NCBI public database. Reads similar to those sequences of *diaph* were extracted by tblastn and manually assembled. Sequences were extended with reads found by blastn with the edge sequence of the first assembles as queries to reconstruct whole-protein coding regions.

### Molecular Phylogenetic Analysis

We carried out molecular phylogenetic analysis of *diaph* using Mrbayes version 3.2.6 (Ronquist et al. 2012). Amino acid sequences were first aligned by ClustalW version 2.1.0 and modified properly considering with exon–intron structure. JTT model was adopted as an evolutionary model according to estimation by MEGA 7.0.9 (Kumar et al. 2016). We reconstructed the basian and maximum likelihood trees of *diaph* based on its whole-protein sequences as well as on FH2 domain sequences by MEGA 7.0.9. To examine the legitimacy of evolutionary loss of one of *diaph* duplicates, we conducted tblastn searches in whole-genome sequence data of *Littorina saxatilis* (SRX4809132) and *Potamopyrgus antipodarum* (SRX2550281). Sequences of DIAPH proteins from *Rapana venosa* (*diaph-Hyp1* and *diaph-Hyp2*), *L. saxatilis* and *P. antipodarum* were used for the query of tblsatn.

### Protein Domain and Exon–Intron Structure Analysis

Domain composition of DIAPH proteins was examined by using Pfam version 32.0 (Finn et al., 2014) as previously described with slight modifications (Noda and Satoh 2008). Exon–intron structure was compared using reconstructed *diaph* transcript sequences and reads of whole-genome sequencing by NGS. All introns’ 5' and 3' splice sites, i.e. intron boundaries, in protein coding regions were identified.

### Formin Family Genes Survey

To examine the extent of evolutionary gene conservation across the animal phyla, gene models of 24 animals and a choanoflagellate as an out group in NCBI database were surveyed by tblastn with Formin family proteins of humans as queries. Annotation of *formin* genes was carried out as previously described with blast bidirectional hit search, Pfam domain analysis and molecular phylogenetic analysis (Noda 2011).

## Results

### Molecular Phylogeny of *Diaph* in Gastropoda

**Fig. 2:**
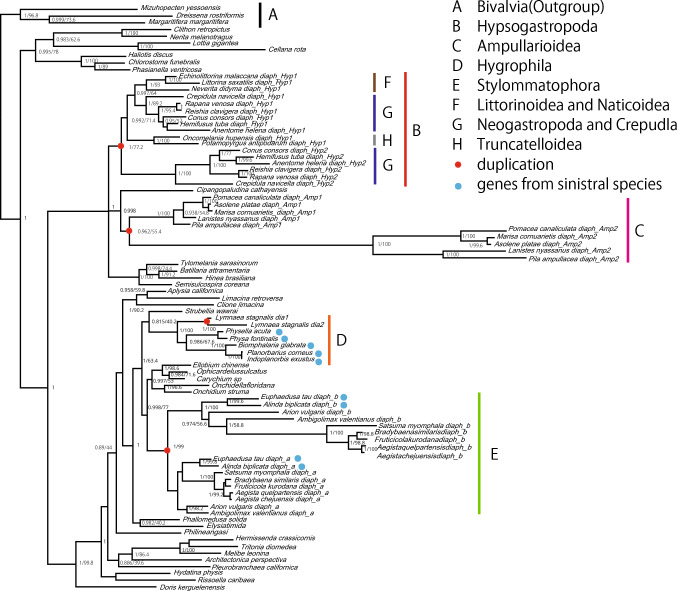
Molecular phylogeny of *diaphanous-related formin* (*diaph*) genes in gastropods, reconstructed by Bayesian method. Red circles show the four independent occurrences of duplication. Blue circles indicate duplicates that are expressed in sinistral taxa. Posterior probabilities higher than 0.8 and bootstrap values of maximum likelihood tree which supports the topologies of bayesian tree are shown on nodes, for which *diaph* sequences of three bivalve species were used as the outgroup

**Fig. 3:**
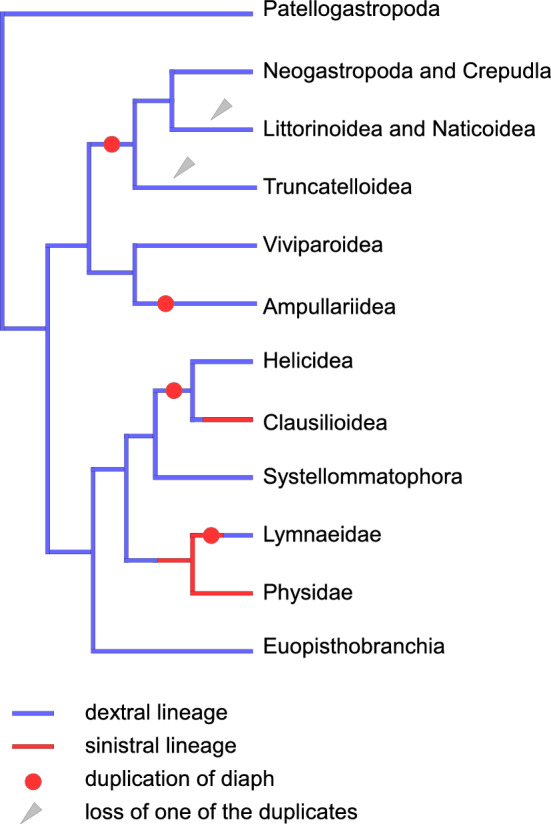
Evolutionary history of *diaph* duplications, duplicate losses and whole-body reversals in gastropod groups that contain taxa with genes examined in this study

Taking advantage of the high quantity and quality RNA and DNA sequence data of molluscs especially gastropods deposited in NCBI, we assembled *diaph*-related sequences from 67 species in 51 families of the phylum Mollusca (Ponder et al. 2020b; Supplementary Table S1). Our results show that all of these molluscs including three bivalves and one cephalopod have at least one *diaph* copy, which is probably highly conserved. We found that two copies of *diaph* are present in the genome of 21 of 63 gastropods included, suggesting the occurrence of duplication during gastropod evolutionary radiation. To substantiate our re-examination of molluscan *diaph* genes and thus to determine evolutionary points where the duplication occurred in the animal tree of gastropods, we established the molecular phylogeny of *diaph* using the entire amino-acid sequences (Fig. 2; Supplementary Fig. S1 for ML tree) and FH2 domain sequences (Fig. S2 for Bayesian inference tree; Fig. S3 for ML tree). Almost the same tree topologies were obtained by the four methods. We plotted *diaph* duplication and loss events detected by this analysis on the phylogeny of gastropod groups (Fig. 3).

### *Diaph* Duplication and Loss

Our *diaph* phylogeny showed that duplications have independently occurred in at least four phylogenetic lineages of the class Gastropoda. Within the Panpulmonata clade, the gene has been duplicated at least twice independently, in the ancestors of the superorder Stylommatophora (*diaph-a* and *diaph-b*) (Fig. 2, clade E) and the hygrophilid family Lymnaeidae (*dia1* and *dia2*) (Fig. 2, a class of within clade D). In addition, two duplications occurred in the ancestors of Hypsogastropoda (*diaph-Hyp1* and *diaph-Hyp2*) (Fig. 2, clade B) and Ampullarioidea (*diaph-Amp1* and *diaph-Amp2*) (Fig. 2, clade C).

One of the duplicates (*diaph-Hyp2*) has been lost twice within the Hypsogastropoda clade; in the ancestor of Truncatelloidea (*Potamopyrgus antipodarum* and *Oncomelania hupensis*) (Fig. 2, clade H) and in that of Littorinoidea and Naticoidea (*Littorina saxatilis*, *Echinolittorina malaccana* and *Neverita didyma*) (Fig. 2, clade F). In theory there is a risk to interpret the technical overlooking of one of those duplicates as the loss in the genome. We thus conducted tblastn search in genome sequencing data from *Littorina saxatilis* and *Potamopyrgus antipodarum*. A plenty of reads were counted for *diaph-Hyp1*. However, no *diaph-Hyp2* like reads were found (Table 1). Our results therefore support the loss of *diaph-Hyp2* in the ancestors of these species.

**Table 1:** Loss of one duplicates of *diaph* gene in genomes of *Littorina saxatilis* and *Potamopyrgus antipodaru*

Species	Gene names	Query sequences			
		*Rapana venosa diaph-Hyp1*	*Rapana venosa diaph-Hyp2*	*L. littorea diaph-Hyp1*	*P. antipodarum diaph-Hyp1*
*Littorina saxatilis*	*diaph-Hyp1*	594	524	695	–*
	*diaph-Hyp2* like	0	0	0	–
	not *diaph*	39	68	93	–
*Potamopyrgus antipodarum*	*diaph-Hyp1*	89	76	–	121
	*diaph-Hyp2* like	0	0	–	0
	not *diaph*	19	22	–	13

^*^not examined.

Loss of one duplicate of *diaph* (*diaph-Hyp2*) was confirmed by the tblastn search. When plenty of reads of duplicate (*diaph-Hyp1*) and no reads of the other (diaph-Hyp2) were found in the genome DNA seq data, loss of the duplicate was supported. The queries for tblastn are shownin the first row. The corresponding genes for the reads are written in the second column. The number of reads hit by tblastn are written in the third to sixth columns

### Reversal Does not Correlate with *Diaph* Duplication or Loss

We found that independent events of *diaph* duplication or loss in Gastropoda accompanied no case of reversal, except one that may have occurred after duplication in the ancestor of freshwater Lymnaeidae. Many lineages of Stylommatophora have retained dextrality after *diaph* became duplicated in their dextral ancestor. Similarly, in Caenogastropoda, *diaph* duplication has preceded in the dextral ancestor of Ampullarioidea as well as in that of Hypsogastropoda, which had been followed by no case of sinistrality evolution.

One of *diaph* duplicated paralogs has been lost at least twice in the clade of hypsogastropods, which also did not accompany the evolution of reversal in the following lineage. Moreover, the two events of reversal in pulmonates independently happened without accompanying *diaph* duplication or loss. The sinistral clade has been derived from the dextral ancestor of freshwater Hygrophila with no duplication. In parallel, in terrestrial Stylommatophora, the sinistrality of Clausiliidae, represented by *Euphaedusa tau* and *Alinda biplicata* in the present study, has evolved with no loss of *diaph* duplicates. These lines of evidence indicate that left–right reversals in Gastropoda have evolved without addition or reduction of the *diaph* paralog.

### Domain Composition and Exon–Intron Structure

**Fig. 4:**
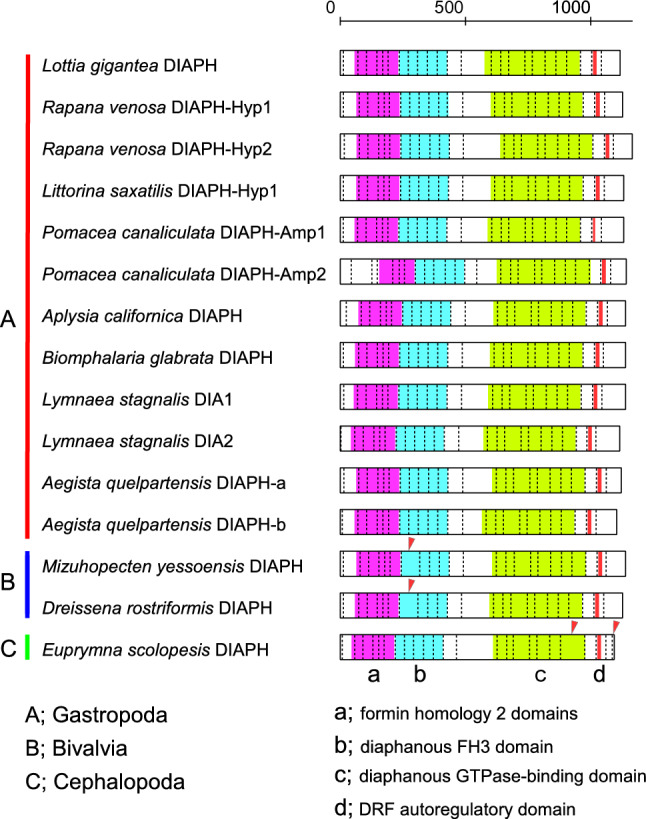
DIAPH protein domain structure in three molluscan classes. Magenta, formin homology 2 domains; cyan, diaphanous FH3 domain; green, diaphanous GTPase-binding domain; and red, DRF autoregulatory domain. Vertical broken lines show exon–intron boundaries of DIAPH in gastropods. Red arrowheads indicate difference in exon–intron boundaries between bivalves/cephalopods and gastropods. The top ruler indicates amino acid positions in the primary sequence

Modification in DIAPH protein domain composition might have been involved in the evolution of left–right reversal. We confirmed that DIAPH protein did not, however, differ in domain composition among three molluscan classes: Gastropoda, Bivalvia and Cephalopoda (Fig. 4). From the N- to C-terminal the protein contains domains of formin homology 2, diaphanous FH3, diaphanous GTPase-binding, and DRF autoregulatory, regardless of the taxon examined. This has similarly been reported for other animals (Schönichen and Geyer 2010).

On the other hand, molluscan *diaph* genes vary in exon–intron structure among the classes. There are 24 introns within the protein coding region of gastropod *diaph*. The bivalve *diaph* contains 23 introns without the one corresponding to the 8th gastropod intron (Fig. 4). Cephalopod genomes lack the intron corresponding to the 21st in gastropods and possess the 25th intron with no correspondence to the gastropod or bivalve introns. However, these changes in intron–exon structure of *diaph* genes are unlikely to affect the domain composition of DIAPH protein (Fig. 4).

### Conservation of *Formin* Family Genes in Metazoans

*Diaph* is a member of one of seven subfamilies that compose the *formin* gene family (Schönichen and Geyer 2010). To assess the relative extent of evolutionary conservation, we examined whether a gene of each subfamily is present in the genome of 24 animal species that represent clades across 14 metazoan phyla, including a sponge (*Amphimedon queenslandica*) and a placozoan (*Trichoplax adhaerens*). The genome of a choanoflagellate *(Monosiga brevicollis*) was used as an outgroup.

**Fig. 5:**
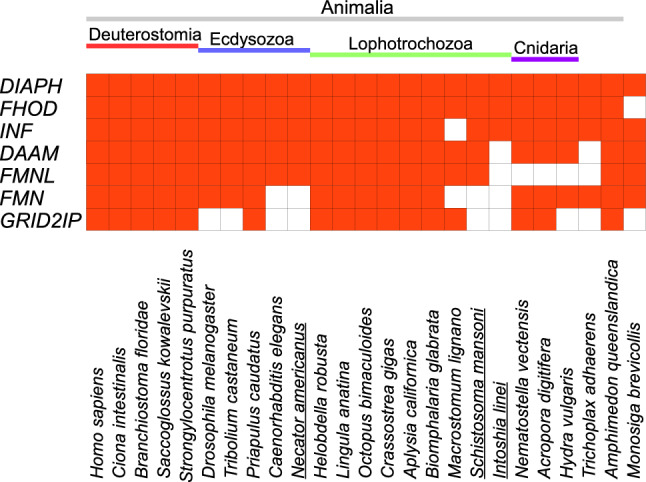
Comparison of evolutionary conservativeness among seven *formin* family genes. Red, deuterostomes; blue, ecdysozoans; green, lophotrochozoans; and purple, cnidarians. Parasitic animals are underlined

We found that the seven gene subfamilies strikingly differ in the extent of evolutionary conservation across these clades of eukaryotes (Fig. 5). The *diaph* subfamily is consistently retained by all the organisms examined. *FHOD* is also commonly present in all the animal genomes including those of ecdysozoan and spiralian parasites (*Necator americanus*, *Schistosoma mansoni*, and *Intoshia linei*). On the other hand, we did not find the subfamilies of *INF* in a platyhelminth (*Macrostomum lignano*) and of *DAAM* in the spiralian parasite or the placozoan. *FMNL* is missing in five animal species of the spiralian, cnidarian and placozoan. Five ecdysozoan and spiralian species also lack *FMN*. The subfamily *GRID2IP* conspicuously differs from the *diaph* subfamily in its absence over nine species, including the ecdysozoan, spiralian, chidarian, placozoan and choanoflagellate. These results show that DIAPH proteins are most thoroughly conserved among the *formin* subfamilies regardless of whether bilateral handedness develops through spiral cleavage.

## Discussion

Our molecular phylogeny of the *diaph* gene disclosed that it has gone through at least four events of duplication and two events of loss after duplication phylogenetically independently from one another in Gastropoda, in which left–right reversed lineages recurrently evolved. We found no sign of evolutionary correlation between *diaph* duplication and whole-body reversal.

Gastropoda radiated from the dextral ancestor with a single *diaph* homolog. Our results show that two independent events of sinistrality evolution by reversal gave rise to sinistral clade including Physidae and Planorbidae in freshwater Hygrophila and the mostly sinistral clausiliid clades in terrestrial Stylommatophora, regardless of whether their dextral ancestors had one or two *diaph* copies. Terrestrial dextral *Bradybaena* and freshwater dextral *Lymnaea* possess two *diaph* copies that originated in different events of duplication within pulmonates. The dextrality of the former represents the ancestral handedness of Gastropoda conserved since before *diaph* duplication, unlike the latter exhibits dextrality that was recovered by reversal from sinistrality.

We demonstrated that DIAPH proteins are expressed in animals of the 14 metazoan phyla as well as in a choanoflagellate. The *diaph* gene only exhibits such a thorough evolutionary conservation among the seven *formin* gene subfamilies. This finding corroborates that DIAPH proteins bare a variety of indispensable functions for cytoskeletal processes generally across eukaryotes, including Deuterostomia and Ecdysozoa which develop bilateral asymmetry in their distinctive modes with no spiral cleavage (Bogdan et al. 2013). In a fruit fly and round worm of the latter clade, *diaph* mutants go through defects in cytokinesis to be lethal (Castrillon and Wasserman 1994; Severson et al. 2002). Similarly, in both dextral *Lymnaea* (Hierck et al. 2005; Asami et al. 2008) and *Bradybaena* (Utsuno and Asami 2010), insufficient maternal expression at only one *diaph* despite having been duplicated produces the reversed progeny that barely survives to the veliger stage (Utsuno et al. 2011; Davison et al. 2016; Kuroda et al. 2016; Noda et al. 2019). These cases exemplify that both of *diaph* duplicates have to be expressed not to be eliminated by internal selection. This indicates that the protein products of both of *diaph* duplicates hold general roles for development as cellular machinery components (Bogdan et al. 2013) other than determining the wild type handedness of spiral cleavage (Martín-Durán & Marlétaz 2020), which gastropods uniquely reversed (Vermeij 1975; Lesoway and Henry 2019; Ponder et al. 2020a).

Sinistrals develop with dextrals in the same uterus in natural populations of a dextral freshwater operculate snail of *Campeloma* with ovoviviparity, but undergo survival selection against after birth (van Cleave 1936). Handedness mutant strains of sinistral ovoviviparous clausiliids *Alinda biplicata* (Degner 1952) and *Euphaedusa tau* (Utsuno et al. 2010) also produce both dextral and sinistral offspring, in which at least the latter strain has no mutation in amino acid sequence or expression of either one of *diaph* duplicates (Noda et al. unpublished data). These lines of currently available knowledge support that *diaph* is neither a primary determinant of spiralian handedness nor has been responsible for the evolution of reversal in ovoviviparous gastropods.

In terrestrial Stylommatophora, all of extant taxa examined possess two *diaph* copies in the genome, regardless of their handedness, indicating that one duplication independently occurred in their dextral ancestor. *Bradybaena similaris* is an example of dextral species in the stylommatophoran family Camaenidae, in which decline of maternal expression of a *diaph* duplicate causes failure in embryonic metamorphosis by and large (Noda et al. 2019). This indicates that the loss-of-function mutation of a *diaph* duplicate could not have been responsible for sinistrality, for which populations have become fixed repeatedly within the Camaenidae clade (Hoso et al. 2010; Chen et al. 2021). In chirally dimorphic *Partula suturalis* of the family Partulidae, there were populations that were fixed for dextrality and those for sinistrality (Johnson et al. 1993). Crossing experiments showed that their sinistrality was dominant to dextrality in maternal inheritance (Murray and Clarke 1976). Thus, the sinistral handedness of this species could not have been the outcome of expression loss of a duplicate gene. Moreover, dextral lineages have also repeatedly evolved by recovering the ancestral handedness within derived sinistral clades, for example in Camaenidae (Ueshima and Asami 2003), Clausiliidae (Gittenberger et al. 2012; Fehér et al. 2013; Páll‐Gergely et al. 2019) and Dyakiidae (Jirapatrasilp et al. 2021). These reversals to the sinistral and back to the dextral in the Stylommatophora could not have occurred repeatedly if the expression loss and recovery of a *diaph* duplicate were responsible for sinistrality and dextrality, respectively.

We therefore conclude that the wild-type sinistrality of gastropods represents a unique example of a recurrently evolved novelty, loss of which recovers the ancestral dextrality, instead of the phenotype resulting from loss-of-function mutation of a *diaph* duplicate. Our findings direct further empirical studies to unravel the genetic key determinant of the wild-type handedness in dextral and sinistral lineages that evolved through population fixation for the reversal of bilateral handedness in Gastropoda.

### Supplementary Information

Below is the link to the electronic supplementary material.

Supplementary file1 (PDF 150 KB)

Supplementary file2 (PDF 223 KB)

Supplementary file3 (PDF 226 KB)

Supplementary file4 (XLS 25 KB)

## Data Availability

Data necessary to re-create the figures can be found on our Unit site: https://osit.jp.group.mgu.
