# Supplementary figures and images for: Left–Right Reversal Recurrently Evolved Regardless of Diaphanous-Related Formin Gene Duplication or Loss in Snails
Source: J Mol Evol. 2023 Sep 25;91(5):721–9. doi: 10.1007/s00239-023-10130-3 (PMC10598177; doi:10.1007/s00239-023-10130-3)

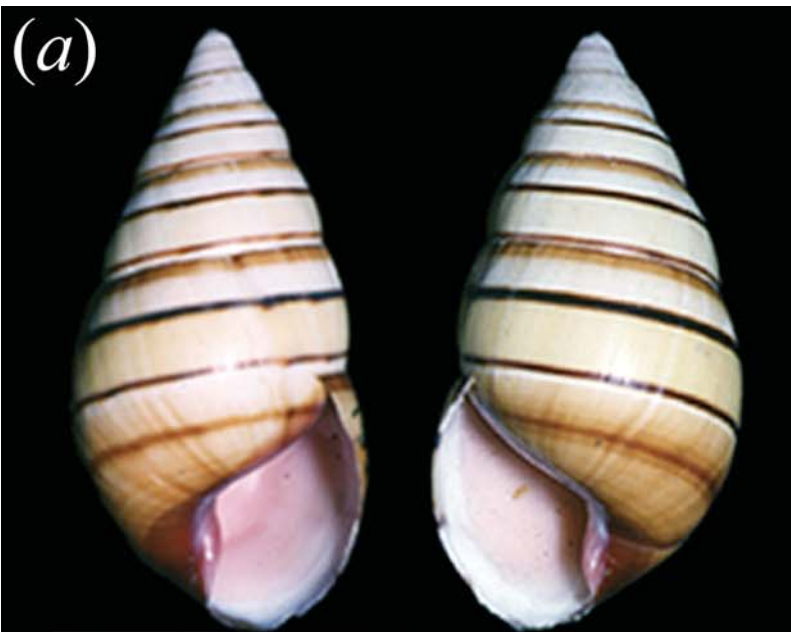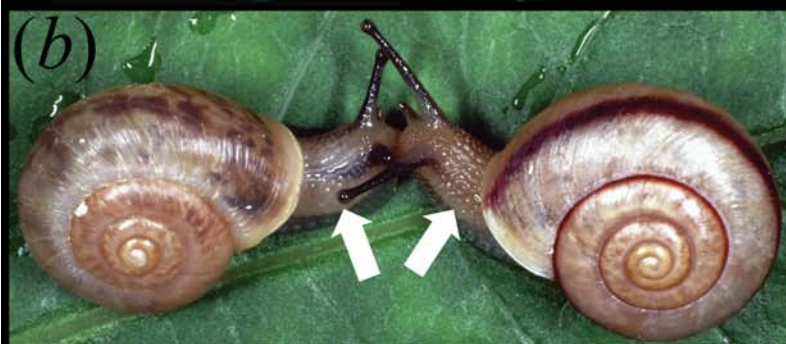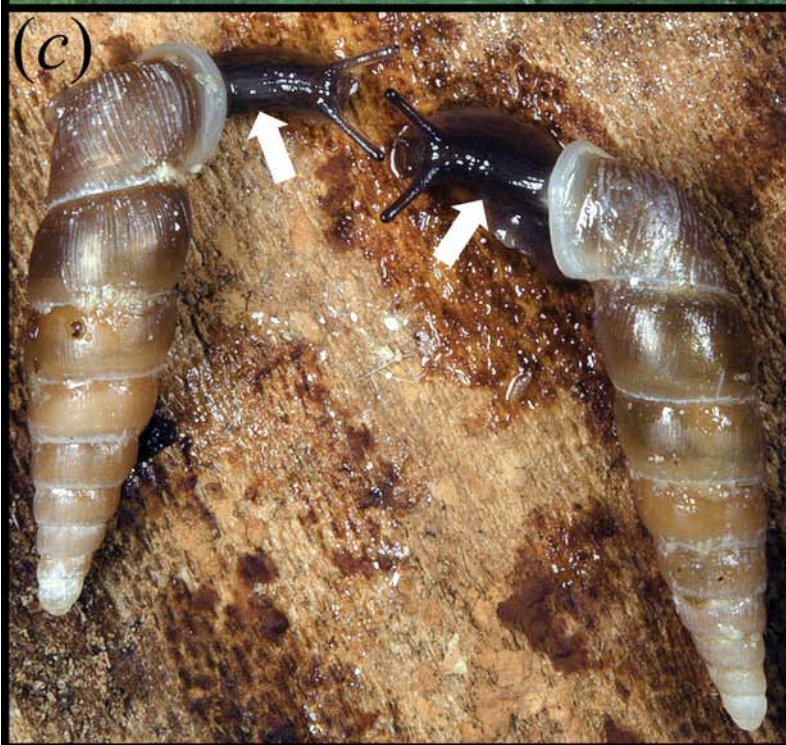

Supplement: Supplementary file 1 — Supplementary file1 (PDF 150 KB) [file 239_2023_10130_MOESM1_ESM.pdf]
