# Supplementary material for: Left–Right Reversal Recurrently Evolved Regardless of Diaphanous-Related Formin Gene Duplication or Loss in Snails
Source: J Mol Evol. 2023 Sep 25;91(5):721–9. doi: 10.1007/s00239-023-10130-3 (PMC10598177; doi:10.1007/s00239-023-10130-3)
Supplement: Supplementary file 2 — Supplementary file2 (PDF 223 KB) [file 239_2023_10130_MOESM2_ESM.pdf]

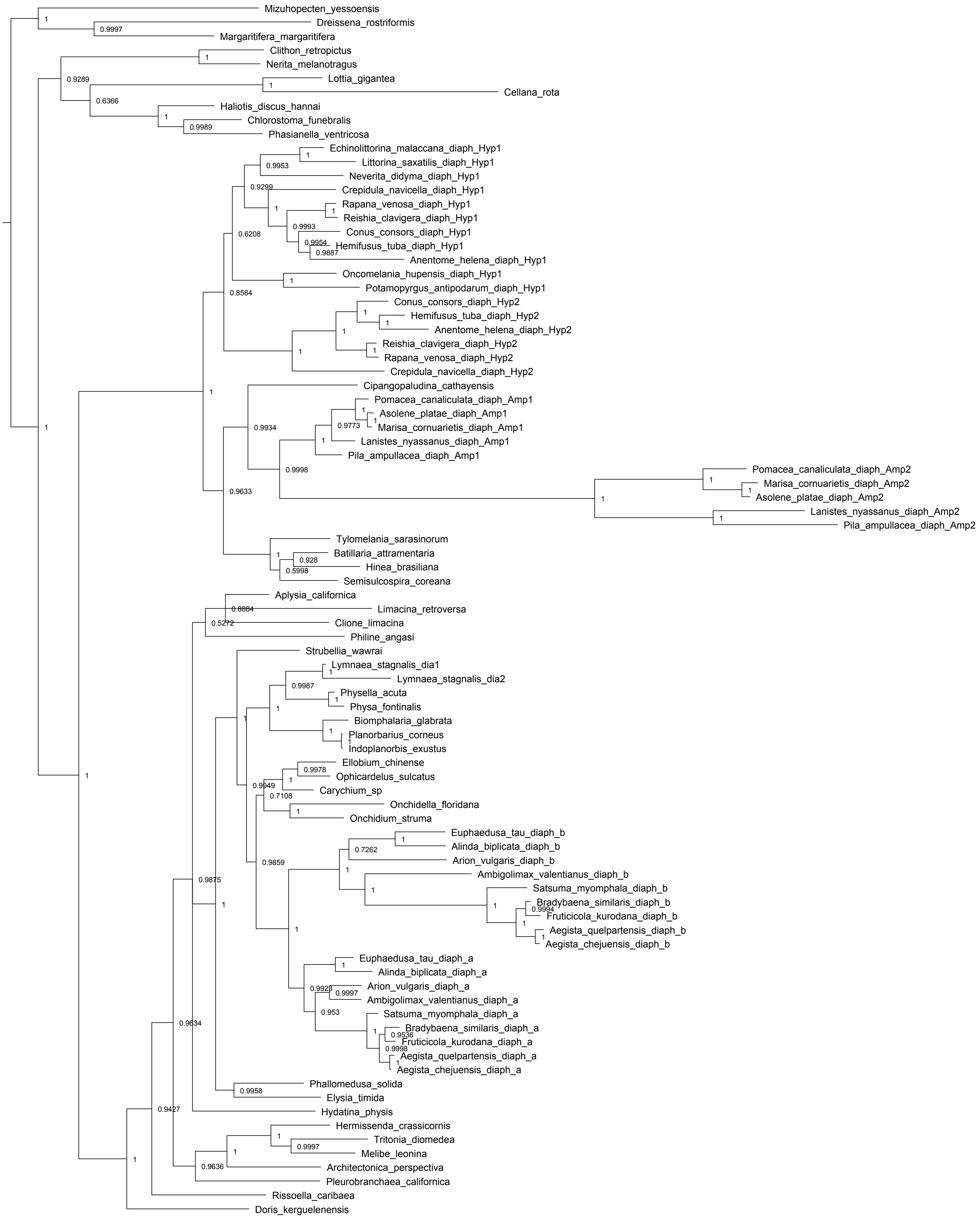

Supplementary Figure S2. Molecular phylogeny of *diaph* genes constructed by Bayesian methods using FH2 domain amino acids. The numbers of node show posterior probabilities.
